# (Di-2-pyridylamine-κ^2^ *N* ^1^,*N* ^1′^)bis­(methacrylato-κ*O*)nickel(II) sesqui­hydrate

**DOI:** 10.1107/S1600536808009008

**Published:** 2008-04-10

**Authors:** Fankun Meng, Lili Xia, Xiaoxiao Zhu

**Affiliations:** a Department of Chemistry and Chemical Engineering, Daqing Normal University, 163712 Daqing, Heilongjiang, People’s Republic of China; b The No. 4 Middle School of Liaocheng, 252000 Liaocheng, Shandong, People’s Republic of China; c Taishan University, 271021 Taian, Shandong, People’s Republic of China

## Abstract

In the title mononuclear complex, [Ni(C_4_H_5_O_2_)_2_(C_10_H_9_N_3_)]·1.5H_2_O, the Ni^II^ ion is in a distorted square-planar coordination environment, formed by two O atoms from two methacrylate ligands and two N atoms from a bis-chelating dipyridylamine ligand. In the crystal structure, inter­molecular O—H⋯O and N—H⋯O hydrogen bonds link complex mol­ecules and water mol­ecules into one-dimensional chains.

## Related literature

For the Cu analog of the title compound, see: Liu, *et al.* (2006 ▶). For related literature, see: Carabias-Martínez *et al.* (2006 ▶); Matsui *et al.* (1997 ▶); Wang *et al.* (1997 ▶); Wu *et al.* (2002 ▶).
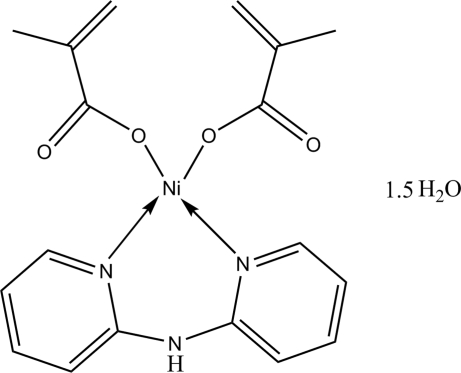


## Experimental

### 

#### Crystal data

[Ni(C_4_H_5_O_2_)_2_(C_10_H_9_N_3_)]·1.5H_2_O
*M* *_r_* = 427.10
Monoclinic, 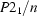

*a* = 8.3686 (9) Å
*b* = 15.7235 (16) Å
*c* = 15.5396 (17) Å
β = 101.483 (2)°
*V* = 2003.8 (4) Å^3^
*Z* = 4
Mo *K*α radiation
μ = 1.00 mm^−1^
*T* = 293 (2) K
0.29 × 0.22 × 0.18 mm

#### Data collection

Bruker SMART CCD area-detector diffractometer
Absorption correction: multi-scan (*SADABS*; Sheldrick, 1996 ▶) *T*_min_ = 0.740, *T*_max_ = 0.872
9960 measured reflections
3512 independent reflections
2720 reflections with *I* > 2σ(*I*)
*R*_int_ = 0.077

#### Refinement

*R*[*F* ^2^ > 2σ(*F* ^2^)] = 0.036
*wR*(*F* ^2^) = 0.084
*S* = 1.00
3512 reflections
311 parameters
13 restraints
H atoms treated by a mixture of independent and constrained refinement
Δρ_max_ = 0.32 e Å^−3^
Δρ_min_ = −0.24 e Å^−3^

### 

Data collection: *SMART* (Bruker, 1996 ▶); cell refinement: *SAINT* (Bruker, 1996 ▶); data reduction: *SAINT*; program(s) used to solve structure: *SHELXS97* (Sheldrick, 2008 ▶); program(s) used to refine structure: *SHELXL97* (Sheldrick, 2008 ▶); molecular graphics: *SHELXTL* (Sheldrick, 2008 ▶); software used to prepare material for publication: *SHELXTL*.

## Supplementary Material

Crystal structure: contains datablocks I, global. DOI: 10.1107/S1600536808009008/lh2600sup1.cif

Structure factors: contains datablocks I. DOI: 10.1107/S1600536808009008/lh2600Isup2.hkl

Additional supplementary materials:  crystallographic information; 3D view; checkCIF report

## Figures and Tables

**Table:** 

Ni1—O3	1.9669 (18)
Ni1—N2	1.980 (2)
Ni1—N1	1.9801 (18)
Ni1—O1	1.9843 (17)

**Table:** 

O3—Ni1—N2	93.16 (8)
O3—Ni1—N1	154.32 (8)
N2—Ni1—N1	92.47 (8)
O3—Ni1—O1	92.24 (8)
N2—Ni1—O1	153.74 (8)
N1—Ni1—O1	93.70 (7)

**Table 2:** Hydrogen-bond geometry (Å, °)

*D*—H⋯*A*	*D*—H	H⋯*A*	*D*⋯*A*	*D*—H⋯*A*
O6—H6*B*⋯O3^i^	0.85	2.31	3.030	143
N3—H19⋯O5^ii^	0.86	1.99	2.837 (3)	170
O5—H20⋯O2	0.96 (3)	1.75 (3)	2.698 (3)	169 (2)
O5—H21⋯O4^iii^	0.92 (3)	1.88 (3)	2.789 (3)	169 (3)

Symmetry codes: (i) 
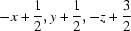
; (ii) 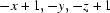; (iii) 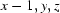.

**Fig. 1.**
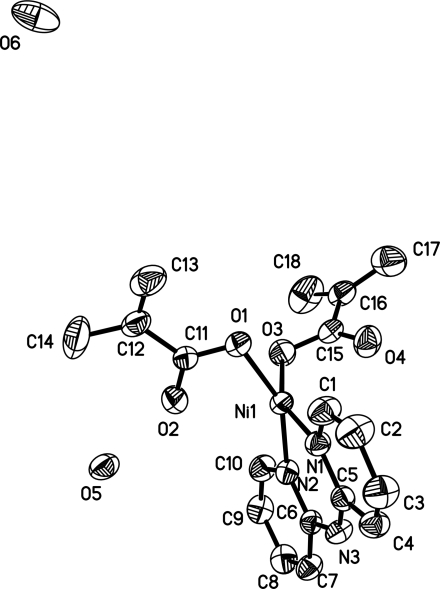
The structure of the title complex, showing 30% probability displacement ellipsoids and the atom-numbering scheme. The H atoms are omitted.

**Fig. 2.**
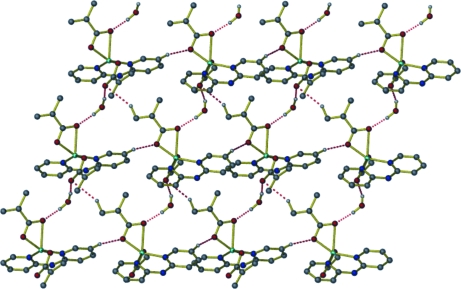
The one-dimensional structure formed via intermolecular O—H···O and N—H···O hydrogen bonds. Hydrogen bonds are shown as red lines.

## supplementary crystallographic information

### Comment

Methacrylic acid and its derivatives are biologically active compounds which are widely used as herbicides and plantgrowth substances (Matsui *et al.*, 1997; Carabias-Martínez *et al.*, 2006). Due to their versatile bonding modes with metal ions, they have also been used in the synthesis of mononuclear or multi-nuclear compounds (Wang *et al.*, 1997; Wu *et al.*, 2002). In order to develop some new topological structures, the reaction system of a nickel(II)chloride with methacrylic acid and dipyridin-2-ylamine has been explored.

Herein we report the structure of the title compound (Fig. 1). It is isostructural with the Cu analog (Liu *et al.*, 2006) but we have located and refined an addtional half of a water solvent molecule. The Ni^II^ ion is in a distorted square-planar coordination environment, formed by two O atoms from two methacrylate ligands and two N atoms from a bis-chelataing dipyridin-2-ylamine ligand. In the crystal structure, intermolecular O—H···O and N—H···O hydrogen bonds link complex molecules and water molecules into a to form one-dimensional chains (Fig. 2).

### Experimental

Methacrylic acid and dipyridin-2-ylamine are commercially available, and they were used without further purification. The reaction was carried out under an air atmoshpere. Methacrylic acid (2 mmol), dipyridin-2-ylamine (1 mmol) and nickel(II)chloride (1 mmol) were added to water and the mixture was stirred for 4 h at 323 K. After cooling to room temperature, the solution was filtered. The solvent was removed from the filtrate under vacuum, and the solid residue was recrystallized from ethanol to form yellow crystals which were suitable for X-Ray diffraction study. Yield, 78%. m.p. 547 K. Analysis, calculated for C_18_H_22_N_3_NiO_5.50_: C 50.62, H 5.19, N 9.84; found: C 50.38, H 5.43, N 9.52. The elemental analyses were performed with a Perkine Elemer PE2400II instrument.

### Refinement

Methyl H atoms were included in calculated positions with C—H = 0.96Å and *U*_iso_(H) = 1.5*U*_eq_(C). H atoms bonded to N3 and O6 were included in calculations with N—H = 0.6 Å, O—H = 0.85Å and *U*_iso_(H) = 1.2*U*_eq_(O,*N*). All other H atoms were refined independently with isotropic displacement parameters. The C—H distances refined to 0.90 (3) - 1.01 (3) Å.

### Crystal data

**Table:**

[Ni(C_4_H_5_O_2_)_2_(C_10_H_9_N_3_)]·1.5H_2_O	*F*_000_ = 892
*M**_r_* = 427.10	*D*_x_ = 1.416 Mg m^−^^3^
Monoclinic, *P*2_1_/*n*	Mo *K*α radiation λ = 0.71073 Å
Hall symbol: -P 2yn	Cell parameters from 4631 reflections
*a* = 8.3686 (9) Å	θ = 1.9–28.3º
*b* = 15.7235 (16) Å	µ = 1.00 mm^−^^1^
*c* = 15.5396 (17) Å	*T* = 293 (2) K
β = 101.483 (2)º	Block, blue
*V* = 2003.8 (4) Å^3^	0.29 × 0.22 × 0.18 mm
*Z* = 4	

### Data collection

**Table:**

Bruker SMART CCD area-detector diffractometer	3512 independent reflections
Radiation source: fine-focus sealed tube	2720 reflections with *I* > 2σ(*I*)
Monochromator: graphite	*R*_int_ = 0.077
*T* = 293(2) K	θ_max_ = 25.0º
φ and ω scans	θ_min_ = 1.9º
Absorption correction: multi-scan(SADABS; Sheldrick, 1996)	*h* = −9→9
*T*_min_ = 0.740, *T*_max_ = 0.872	*k* = −18→17
9960 measured reflections	*l* = −15→18

### Refinement

**Table:**

Refinement on *F*^2^	Secondary atom site location: difference Fourier map
Least-squares matrix: full	Hydrogen site location: inferred from neighbouring sites
*R*[*F*^2^ > 2σ(*F*^2^)] = 0.036	H atoms treated by a mixture of independent and constrained refinement
*wR*(*F*^2^) = 0.084	*w* = 1/[σ^2^(*F*_o_^2^) + (0.03*P*)^2^] where *P* = (*F*_o_^2^ + 2*F*_c_^2^)/3
*S* = 1.00	(Δ/σ)_max_ = 0.013
3512 reflections	Δρ_max_ = 0.32 e Å^−^^3^
311 parameters	Δρ_min_ = −0.24 e Å^−^^3^
13 restraints	Extinction correction: none
Primary atom site location: structure-invariant direct methods	

### Special details

**Table:**

Geometry. All e.s.d.'s (except the e.s.d. in the dihedral angle between two l.s. planes) are estimated using the full covariance matrix. The cell e.s.d.'s are taken into account individually in the estimation of e.s.d.'s in distances, angles and torsion angles; correlations between e.s.d.'s in cell parameters are only used when they are defined by crystal symmetry. An approximate (isotropic) treatment of cell e.s.d.'s is used for estimating e.s.d.'s involving l.s. planes.
Refinement. Refinement of *F*^2^ against ALL reflections. The weighted *R*-factor *wR* and goodness of fit *S* are based on *F*^2^, conventional *R*-factors *R* are based on *F*, with *F* set to zero for negative *F*^2^. The threshold expression of *F*^2^ > σ(*F*^2^) is used only for calculating *R*-factors(gt) *etc*. and is not relevant to the choice of reflections for refinement. *R*-factors based on *F*^2^ are statistically about twice as large as those based on *F*, and *R*- factors based on ALL data will be even larger.

### Fractional atomic coordinates and isotropic or equivalent isotropic displacement parameters (Å^2^)

**Table:**

	*x*	*y*	*z*	*U*_iso_*/*U*_eq_	Occ. (<1)
Ni1	0.71669 (3)	0.166235 (18)	0.635377 (19)	0.05233 (13)	
O1	0.6953 (2)	0.18663 (11)	0.75859 (11)	0.0676 (5)	
O2	0.4520 (2)	0.16405 (13)	0.68304 (14)	0.0869 (6)	
O3	0.6959 (2)	0.28811 (11)	0.60684 (13)	0.0744 (5)	
O4	0.9584 (2)	0.26650 (12)	0.62783 (14)	0.0866 (6)	
O5	0.1555 (3)	0.13886 (13)	0.58041 (14)	0.0871 (6)	
O6	0.1576 (18)	0.8401 (6)	0.9099 (9)	0.365 (9)	0.50
H6A	0.2379	0.8070	0.9103	0.437*	0.50
H6B	0.0724	0.8139	0.8846	0.437*	0.50
N1	0.8388 (2)	0.05866 (11)	0.66380 (12)	0.0554 (5)	
N2	0.6341 (2)	0.13116 (12)	0.51218 (13)	0.0555 (5)	
N3	0.7837 (2)	0.00393 (12)	0.51996 (13)	0.0599 (5)	
H19	0.8113	−0.0357	0.4877	0.072*	
C1	0.9209 (4)	0.0486 (2)	0.74736 (19)	0.0786 (8)	
C2	1.0198 (4)	−0.0173 (2)	0.7755 (2)	0.0922 (10)	
C3	1.0384 (4)	−0.0799 (2)	0.7157 (2)	0.0857 (9)	
C4	0.9597 (3)	−0.07131 (17)	0.63130 (19)	0.0682 (7)	
C5	0.8605 (3)	−0.00130 (14)	0.60641 (16)	0.0530 (6)	
C6	0.6718 (3)	0.05967 (14)	0.47463 (15)	0.0514 (5)	
C7	0.5986 (3)	0.03930 (18)	0.38840 (17)	0.0659 (7)	
C8	0.4874 (4)	0.0926 (2)	0.34175 (19)	0.0745 (8)	
C9	0.4470 (4)	0.1665 (2)	0.38006 (19)	0.0724 (8)	
C10	0.5214 (3)	0.18320 (18)	0.4632 (2)	0.0693 (7)	
C11	0.5437 (3)	0.18002 (14)	0.75381 (19)	0.0612 (7)	
C12	0.4763 (4)	0.18975 (16)	0.8346 (2)	0.0745 (8)	
C13	0.5788 (7)	0.1974 (2)	0.9112 (3)	0.1023 (12)	
C14	0.3001 (5)	0.1899 (3)	0.8252 (3)	0.1299 (14)	
H13	0.2721	0.2052	0.8801	0.195*	
H11	0.2583	0.1342	0.8081	0.195*	
H12	0.2534	0.2304	0.7810	0.195*	
C15	0.8403 (4)	0.31425 (16)	0.60955 (16)	0.0606 (6)	
C16	0.8616 (4)	0.40609 (18)	0.58992 (18)	0.0745 (7)	
C17	1.0179 (7)	0.4399 (3)	0.6099 (3)	0.1124 (13)	
C18	0.7233 (5)	0.4528 (2)	0.5499 (3)	0.1287 (14)	
H17	0.7563	0.5083	0.5343	0.193*	
H18	0.6504	0.4584	0.5901	0.193*	
H16	0.6688	0.4237	0.4980	0.193*	
H1	0.913 (3)	0.0917 (18)	0.7842 (18)	0.083 (9)*	
H2	1.086 (3)	−0.0193 (18)	0.8329 (14)	0.099 (10)*	
H3	1.108 (4)	−0.131 (2)	0.733 (2)	0.117 (11)*	
H4	0.964 (3)	−0.1118 (14)	0.5870 (14)	0.066 (7)*	
H5	0.630 (2)	−0.0103 (11)	0.3650 (14)	0.058 (7)*	
H6	0.430 (3)	0.0750 (19)	0.281 (2)	0.106 (10)*	
H7	0.373 (3)	0.2059 (16)	0.3503 (17)	0.076 (8)*	
H8	0.495 (3)	0.2368 (17)	0.4916 (17)	0.084 (8)*	
H9	0.690 (2)	0.187 (2)	0.919 (2)	0.118 (16)*	
H10	0.523 (5)	0.207 (2)	0.958 (3)	0.153 (15)*	
H14	1.099 (4)	0.403 (2)	0.633 (2)	0.133 (17)*	
H15	1.031 (4)	0.4937 (15)	0.592 (2)	0.134 (14)*	
H20	0.257 (3)	0.1554 (17)	0.6173 (18)	0.099 (11)*	
H21	0.082 (4)	0.1753 (18)	0.598 (2)	0.128 (14)*	

### Atomic displacement parameters (Å^2^)

**Table:**

	*U*^11^	*U*^22^	*U*^33^	*U*^12^	*U*^13^	*U*^23^
Ni1	0.0512 (2)	0.0530 (2)	0.0534 (2)	0.00317 (13)	0.01212 (15)	−0.00762 (14)
O1	0.0629 (12)	0.0792 (12)	0.0624 (11)	−0.0013 (9)	0.0162 (9)	−0.0175 (9)
O2	0.0608 (12)	0.1171 (17)	0.0832 (14)	−0.0144 (11)	0.0152 (11)	−0.0286 (12)
O3	0.0689 (12)	0.0613 (11)	0.0941 (13)	0.0041 (9)	0.0191 (10)	−0.0042 (10)
O4	0.0766 (13)	0.0790 (13)	0.1072 (16)	0.0182 (10)	0.0254 (12)	0.0057 (11)
O5	0.0704 (13)	0.0955 (14)	0.0905 (15)	0.0182 (11)	0.0041 (12)	−0.0320 (12)
O6	0.410 (19)	0.215 (11)	0.395 (19)	−0.049 (10)	−0.096 (16)	0.069 (10)
N1	0.0556 (12)	0.0580 (12)	0.0519 (11)	0.0019 (9)	0.0089 (10)	−0.0038 (9)
N2	0.0527 (12)	0.0598 (12)	0.0546 (12)	0.0037 (10)	0.0121 (10)	0.0011 (10)
N3	0.0686 (13)	0.0582 (12)	0.0528 (12)	0.0107 (10)	0.0121 (10)	−0.0075 (10)
C1	0.084 (2)	0.086 (2)	0.0597 (18)	0.0167 (17)	−0.0003 (15)	−0.0109 (16)
C2	0.096 (2)	0.105 (2)	0.0633 (19)	0.030 (2)	−0.0122 (18)	−0.0020 (18)
C3	0.088 (2)	0.078 (2)	0.084 (2)	0.0213 (17)	−0.0027 (18)	0.0054 (18)
C4	0.0713 (17)	0.0595 (16)	0.0708 (18)	0.0055 (13)	0.0066 (15)	−0.0046 (14)
C5	0.0500 (13)	0.0518 (13)	0.0579 (15)	−0.0019 (11)	0.0125 (11)	0.0015 (12)
C6	0.0490 (13)	0.0567 (14)	0.0504 (13)	−0.0009 (11)	0.0146 (11)	0.0006 (11)
C7	0.0716 (17)	0.0738 (18)	0.0531 (16)	0.0097 (14)	0.0147 (14)	−0.0050 (14)
C8	0.0739 (19)	0.098 (2)	0.0507 (16)	−0.0005 (17)	0.0103 (15)	0.0084 (16)
C9	0.0701 (19)	0.081 (2)	0.0639 (18)	0.0116 (16)	0.0085 (15)	0.0116 (16)
C10	0.0681 (18)	0.0674 (18)	0.0710 (19)	0.0138 (14)	0.0101 (15)	0.0034 (14)
C11	0.0621 (17)	0.0529 (15)	0.0728 (18)	−0.0060 (12)	0.0234 (15)	−0.0125 (12)
C12	0.104 (2)	0.0500 (14)	0.0812 (19)	−0.0052 (14)	0.0472 (18)	−0.0095 (14)
C13	0.155 (4)	0.084 (2)	0.081 (2)	−0.020 (3)	0.056 (3)	−0.0093 (19)
C14	0.119 (3)	0.144 (3)	0.153 (4)	0.010 (2)	0.089 (3)	0.005 (3)
C15	0.0705 (18)	0.0624 (16)	0.0520 (14)	0.0061 (14)	0.0193 (14)	−0.0061 (12)
C16	0.101 (2)	0.0637 (17)	0.0669 (17)	−0.0049 (15)	0.0361 (16)	−0.0069 (14)
C17	0.147 (4)	0.093 (3)	0.104 (3)	−0.034 (3)	0.044 (3)	0.000 (2)
C18	0.160 (3)	0.080 (2)	0.161 (4)	0.039 (2)	0.067 (3)	0.030 (2)

### Geometric parameters (Å, °)

**Table:**

Ni1—O3	1.9669 (18)	C4—H4	0.944 (16)
Ni1—N2	1.980 (2)	C6—C7	1.395 (3)
Ni1—N1	1.9801 (18)	C7—C8	1.351 (4)
Ni1—O1	1.9843 (17)	C7—H5	0.921 (15)
O1—C11	1.260 (3)	C8—C9	1.378 (4)
O2—C11	1.235 (3)	C8—H6	1.01 (3)
O3—C15	1.269 (3)	C9—C10	1.343 (4)
O4—C15	1.228 (3)	C9—H7	0.93 (3)
O5—H20	0.961 (18)	C10—H8	1.00 (3)
O5—H21	0.921 (18)	C11—C12	1.483 (4)
O6—H6A	0.8498	C12—C13	1.327 (5)
O6—H6B	0.8497	C12—C14	1.453 (4)
N1—C5	1.335 (3)	C13—H9	0.930 (18)
N1—C1	1.352 (3)	C13—H10	0.95 (4)
N2—C6	1.333 (3)	C14—H13	0.9600
N2—C10	1.361 (3)	C14—H11	0.9600
N3—C6	1.371 (3)	C14—H12	0.9600
N3—C5	1.371 (3)	C15—C16	1.494 (4)
N3—H19	0.8600	C16—C17	1.389 (5)
C1—C2	1.344 (4)	C16—C18	1.407 (4)
C1—H1	0.90 (3)	C17—H14	0.911 (18)
C2—C3	1.383 (4)	C17—H15	0.906 (19)
C2—H2	0.954 (18)	C18—H17	0.9600
C3—C4	1.353 (4)	C18—H18	0.9600
C3—H3	1.00 (3)	C18—H16	0.9600
C4—C5	1.387 (3)		
			
O3—Ni1—N2	93.16 (8)	C7—C8—C9	119.6 (3)
O3—Ni1—N1	154.32 (8)	C7—C8—H6	118.8 (17)
N2—Ni1—N1	92.47 (8)	C9—C8—H6	121.5 (17)
O3—Ni1—O1	92.24 (8)	C10—C9—C8	118.3 (3)
N2—Ni1—O1	153.74 (8)	C10—C9—H7	118.8 (16)
N1—Ni1—O1	93.70 (7)	C8—C9—H7	122.9 (16)
C11—O1—Ni1	102.29 (16)	C9—C10—N2	123.9 (3)
C15—O3—Ni1	105.48 (16)	C9—C10—H8	119.4 (15)
H20—O5—H21	103 (3)	N2—C10—H8	116.7 (15)
H6A—O6—H6B	107.1	O2—C11—O1	120.6 (2)
C5—N1—C1	116.6 (2)	O2—C11—C12	120.0 (3)
C5—N1—Ni1	125.95 (16)	O1—C11—C12	119.3 (3)
C1—N1—Ni1	117.08 (18)	C13—C12—C14	123.5 (4)
C6—N2—C10	117.4 (2)	C13—C12—C11	118.8 (3)
C6—N2—Ni1	126.19 (16)	C14—C12—C11	117.7 (3)
C10—N2—Ni1	116.32 (18)	C12—C13—H9	124 (2)
C6—N3—C5	132.90 (19)	C12—C13—H10	112 (3)
C6—N3—H19	113.5	H9—C13—H10	124 (4)
C5—N3—H19	113.5	C12—C14—H13	109.5
C2—C1—N1	124.4 (3)	C12—C14—H11	109.5
C2—C1—H1	119.6 (17)	H13—C14—H11	109.5
N1—C1—H1	115.9 (18)	C12—C14—H12	109.5
C1—C2—C3	118.4 (3)	H13—C14—H12	109.5
C1—C2—H2	122.5 (18)	H11—C14—H12	109.5
C3—C2—H2	118.8 (17)	O4—C15—O3	121.8 (2)
C4—C3—C2	118.8 (3)	O4—C15—C16	121.0 (3)
C4—C3—H3	119 (2)	O3—C15—C16	117.2 (2)
C2—C3—H3	122 (2)	C17—C16—C18	123.6 (4)
C3—C4—C5	119.9 (3)	C17—C16—C15	118.1 (3)
C3—C4—H4	123.9 (15)	C18—C16—C15	118.3 (3)
C5—C4—H4	116.1 (15)	C16—C17—H14	116 (3)
N1—C5—N3	120.8 (2)	C16—C17—H15	117 (3)
N1—C5—C4	121.9 (2)	H14—C17—H15	126 (4)
N3—C5—C4	117.3 (2)	C16—C18—H17	109.5
N2—C6—N3	120.8 (2)	C16—C18—H18	109.5
N2—C6—C7	121.1 (2)	H17—C18—H18	109.5
N3—C6—C7	118.1 (2)	C16—C18—H16	109.5
C8—C7—C6	119.8 (3)	H17—C18—H16	109.5
C8—C7—H5	122.3 (14)	H18—C18—H16	109.5
C6—C7—H5	117.9 (14)		
			
O3—Ni1—O1—C11	90.93 (15)	C6—N3—C5—C4	175.2 (2)
N2—Ni1—O1—C11	−10.8 (3)	C3—C4—C5—N1	−0.4 (4)
N1—Ni1—O1—C11	−114.07 (15)	C3—C4—C5—N3	−179.9 (3)
N2—Ni1—O3—C15	−105.33 (17)	C10—N2—C6—N3	−179.7 (2)
N1—Ni1—O3—C15	−2.9 (3)	Ni1—N2—C6—N3	−3.6 (3)
O1—Ni1—O3—C15	100.38 (17)	C10—N2—C6—C7	0.1 (3)
O3—Ni1—N1—C5	−94.3 (2)	Ni1—N2—C6—C7	176.25 (17)
N2—Ni1—N1—C5	8.28 (19)	C5—N3—C6—N2	9.6 (4)
O1—Ni1—N1—C5	162.74 (18)	C5—N3—C6—C7	−170.3 (2)
O3—Ni1—N1—C1	78.9 (3)	N2—C6—C7—C8	0.0 (4)
N2—Ni1—N1—C1	−178.6 (2)	N3—C6—C7—C8	179.8 (2)
O1—Ni1—N1—C1	−24.1 (2)	C6—C7—C8—C9	−0.3 (4)
O3—Ni1—N2—C6	151.52 (18)	C7—C8—C9—C10	0.5 (4)
N1—Ni1—N2—C6	−3.41 (19)	C8—C9—C10—N2	−0.4 (4)
O1—Ni1—N2—C6	−106.9 (2)	C6—N2—C10—C9	0.1 (4)
O3—Ni1—N2—C10	−32.28 (19)	Ni1—N2—C10—C9	−176.4 (2)
N1—Ni1—N2—C10	172.79 (18)	Ni1—O1—C11—O2	0.2 (3)
O1—Ni1—N2—C10	69.3 (3)	Ni1—O1—C11—C12	178.91 (18)
C5—N1—C1—C2	−0.7 (4)	O2—C11—C12—C13	172.6 (3)
Ni1—N1—C1—C2	−174.4 (3)	O1—C11—C12—C13	−6.1 (4)
N1—C1—C2—C3	−0.7 (5)	O2—C11—C12—C14	−7.1 (4)
C1—C2—C3—C4	1.6 (5)	O1—C11—C12—C14	174.2 (3)
C2—C3—C4—C5	−1.1 (5)	Ni1—O3—C15—O4	−0.5 (3)
C1—N1—C5—N3	−179.3 (2)	Ni1—O3—C15—C16	179.79 (18)
Ni1—N1—C5—N3	−6.1 (3)	O4—C15—C16—C17	−12.7 (4)
C1—N1—C5—C4	1.2 (4)	O3—C15—C16—C17	167.0 (3)
Ni1—N1—C5—C4	174.35 (18)	O4—C15—C16—C18	165.4 (3)
C6—N3—C5—N1	−4.3 (4)	O3—C15—C16—C18	−14.9 (4)

### Hydrogen-bond geometry (Å, °)

**Table:**

*D*—H···*A*	*D*—H	H···*A*	*D*···*A*	*D*—H···*A*
O6—H6B···O2^i^	0.85	2.57	3.172	129
O6—H6B···O3^i^	0.85	2.31	3.030	143
N3—H19···O5^ii^	0.86	1.99	2.837 (3)	170
O5—H20···O2	0.96 (3)	1.75 (3)	2.698 (3)	169 (2)
O5—H21···O4^iii^	0.92 (3)	1.88 (3)	2.789 (3)	169 (3)

Symmetry codes: (i) −*x*+1/2, *y*+1/2, −*z*+3/2; (ii) −*x*+1, −*y*, −*z*+1; (iii) *x*−1, *y*, *z*.
